# Targeted Hepatic Delivery of Bioactive Molecules via Nanovesicles: Recent Developments and Emerging Directions

**DOI:** 10.3390/jpm16010001

**Published:** 2025-12-19

**Authors:** Alessia Rita Canestrale, Sharad Kholia, Veronica Dimuccio, Maria Beatriz Herrera Sanchez

**Affiliations:** 1 Department of Medical Sciences, University of Torino, 10126 Turin, Italy; alessiarita.canestrale@unito.it; 2 Molecular Biotechnology Centre, University of Torino, 10126 Turin, Italy; sharadkumar.kholia@unito.it; 3 Fondazione per la Ricerca Biomedica—ONLUS, University of Torino, 10126 Turin, Italy; veronica.dimuccio@unito.it; 4 2i3T, Società per la Gestione dell’incubatore di Imprese e per il Trasferimento Tecnologico, University of Torino, 10135 Turin, Italy

**Keywords:** nanovesicles, extracellular vesicles, lipid nanoparticles, liposomes, nanobubbles, mRNA delivery, liver genetic diseases

## Abstract

Liver diseases, including fibrosis, viral hepatitis, hepatocellular carcinoma, and monogenic genetic disorders, represent a major global health burden with limited therapeutic options and frequent systemic toxicity from conventional treatments. Nanovesicle-based drug and gene delivery systems offer targeted approaches that may improve therapeutic precision and reduce off-target effects. This review aims to evaluate the promise and comparative potential of three key nanovesicle platforms—lipid nanoparticles (LNPs), extracellular vesicles (EVs) and liposomes—for drug and gene delivery in liver disease therapy. A systematic search of peer-reviewed studies published in electronic databases was performed, focusing on preclinical and clinical research investigating the use of LNPs, EVs and liposomes for hepatic drug or gene delivery. Studies were analyzed for vesicle composition, targeting efficiency, payload capacity, therapeutic outcomes, and reported limitations. The analysis indicates that LNPs demonstrate strong efficiency in nucleic acid encapsulation and delivery, supported by growing clinical translation. EVs show promising biocompatibility and innate targeting to hepatic cells but face challenges in large-scale production and standardization. Liposomes remain versatile and well-characterized platforms capable of carrying diverse therapeutic molecules, though rapid clearance can limit their efficacy. Together, these nanovesicle systems hold considerable potential for advancing targeted drug and gene therapies in liver disease. Future work should focus on improving stability, manufacturing scalability, and cell-specific targeting to support clinical translation.

## 1. Introduction

Early investigations of evidence supporting the view that living cells were surrounded by lipid material was reported by Davson et al. in the 1930s [1]. However, the foundational understanding of lipid-based delivery systems can be traced back to the mid-1960s, when studies revealed that phosphatidylcholine from egg yolk could self-assemble in water to form multilamellar vesicles composed of concentric lipid bilayers enclosing portions of the aqueous phase [2]. This finding strongly supported the concept that lipid membranes function as selective barriers between intracellular and extracellular environments, and it encouraged extensive biophysical investigations aimed at elucidating lipid behavior in membrane-like structures. As researchers developed controlled methods for preparing liposomes, defined as aqueous dispersions of nano to microscale lipid bilayer vesicles, they began characterizing properties such as membrane fluidity, permeability, phase transitions, and fusogenic potential [3]. By the late 1970s, attention shifted toward using these vesicles to encapsulate and deliver nucleic acids. Early work demonstrated that mRNA could be loaded into liposomes and remain functionally competent, enabling protein expression in cultured cells, while negatively charged liposomes were shown to facilitate delivery of viral DNA [4,5]. Subsequent in vivo studies in the early 1980s reported expression of plasmid DNA encoding rat pre-proinsulin following intravenous administration of liposomal formulations [6]. Despite this progress, challenges such as limited encapsulation efficiency, low expression levels, and inadequate scalability constrained progress. A major conceptual advance occurred in 1987, when Felgner and colleagues proposed using positively charged lipids to enhance electrostatic interactions with nucleic acids [7]. This led to the synthesis of cationic lipids such as DOTMA, which, when formulated with helper lipids like DOPC or DOPE, generated stable, nanoscale vesicles capable of efficiently transfecting plasmid DNA and mRNA into mammalian cells without requiring additional chemical modification [8]. Their versatility allows encapsulation of both hydrophilic and hydrophobic compounds, and they continue to be used in drug delivery.

The study of extracellular vesicles (EVs) began in 1967 when Peter Wolf observed what he called “platelet dust”, tiny membranous fragments released from activated platelets that were visible under the electron microscope [9]. These particles were initially dismissed as waste by-products of cell damage or activation, as their biological relevance was uncertain. Yet, these structures retained procoagulant activity and carried elements of the parent platelet membrane, suggesting that they might serve more than a passive role in circulation. What appeared to be “dust” would, in time, become recognized as the first sight of a universal biological mechanism.

For nearly twenty years, the concept of extracellular vesicles remained on the margins of cell biology, referenced mainly in hematology and immunology contexts, but the scenario was about to change. In 2007, a new study by Valadi and colleagues demonstrated that exosomes derived from mast cells contained both messenger RNAs and microRNAs capable of being transferred to recipient cells, where they could be translated into functional proteins [10]. This was a remarkable discovery that cells secrete both molecular signals and fragments of their genetic code. The following year, Skog and co-workers showed that glioblastoma-derived microvesicles carry oncogenic mRNA and proteins that promote tumor growth while also being detectable in patient plasma [11]. Thanks to these discoveries, the perception of EVs shifted toward viewing them as active mediators of biological information and key mediators of cell-to-cell communication. These findings carry the conclusions that EVs could function as natural nanocarriers of information, modulating gene expression and influencing distant cells in ways that had previously been attributed only to soluble factors such as cytokines or hormones.

One of the key advantages of EVs lies in their capacity to act as targeted delivery shuttles, enabling the selective transport of therapeutic cargo to specific tissues while minimizing off-target effects and reducing toxicity to healthy tissues [12]. Furthermore, EVs can evade immune surveillance, thereby protecting their cargo from premature degradation, and can also be engineered to release their contents in response to specific physiological or pathological stimuli [12].

These unique properties make EVs particularly attractive for targeting the liver, an organ with high vascularization, efficient endocytosis capacity, and central importance in many metabolic and genetic diseases. In this context, EVs have promising possibilities for the treatment of hepatic disorders, including inherited metabolic conditions such as urea cycle defects, by enabling the delivery of therapeutic molecules directly to hepatocytes in a safe and efficient manner. In parallel with mammalian-derived EVs, plant-derived nanovesicles (PDVs) are increasingly gaining interest as safe and scalable delivery platforms. Derived from and not limited to sources such as grapefruit, ginger, or orange, PDVs are naturally resistant to digestive degradation, can be administered orally, and exhibit minimal immunogenicity, offering a cost-effective alternative to cell-derived EVs [13,14,15]. Their accessibility, stability, and safety profile highlight PDVs as promising candidates for both nutraceutical and therapeutic applications, potentially expanding the range of nanovesicle-based strategies for liver-targeted interventions.

More recently, synthetic systems such as LNPs have already demonstrated clinical success, particularly in delivering mRNA and siRNA to the liver. Their high transfection efficiency and scalable manufacturing made them the foundation of RNA vaccines and other nucleic acid-based therapies. However, their clinical utility is constrained by strong liver tropism, innate immune activation, and limited tolerance to repeated dosing [16,17]. Despite these limitations, continued optimization of LNP formulations, particularly through next-generation ionizable lipids and ligand-based targeting, has led to meaningful improvements in hepatocyte-specific delivery and safety profiles. For example, GalNAc-conjugation allows selective engagement of the asialoglycoprotein receptor (ASGPR), thereby increasing uptake by liver parenchymal cells, while reducing off-target exposure [18]. Moreover, advances in endosomal escape chemistry and lipid architecture continue to enhance intracellular release efficiency, a key determinant of therapeutic potency.

At the same time, there is growing recognition that synthetic vesicles alone may not fully address the complexity of immune interactions and biodistribution restrictions inherent to nucleic acid delivery. Repeated dosing of LNPs, particularly in chronic conditions, can trigger innate immune pathways via pattern-recognition receptors, potentially reducing both tolerability and therapeutic durability [19]. These challenges underline the need for complementary delivery systems capable of achieving sustained or highly targeted effects in vivo.

In this context, nanovesicles have emerged as alternatives to synthetic formulations. Their endogenous membrane composition, natural signaling properties, and capacity for cell-specific communication may offer advantages in immune tolerance, biodistribution, and long-term compatibility. Additionally, their ability to carry diverse molecular cargos, including proteins, lipids, and nucleic acids, positions them as promising candidates for next-generation liver-targeted therapies.

## 2. Nanovesicles

### 2.1. Sources

**Figure 1:**
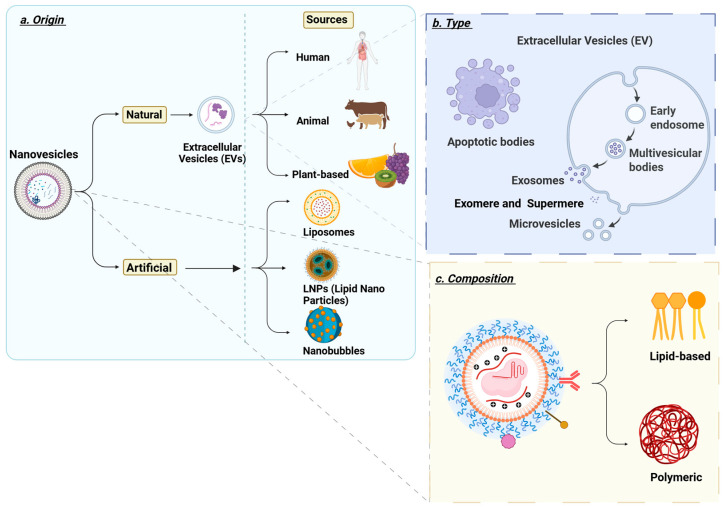
Schematic Classification of Nanovesicles by Origin (**a**), Type (**b**), and Composition (**c**).

Nanovesicles are nanoscale, membrane-bound carriers capable of transporting therapeutic molecules such as drugs, RNA, and proteins within the body. They can be engineered from synthetic or biological materials, allowing for controlled delivery and reduced systemic side effects. Due to their small size and ability to target specific tissues, nanovesicles such as lipid nanoparticles, extracellular vesicles and liposomes are increasingly explored for precision therapy in liver diseases, and they can be classified as shown in Figure 1.

### 2.2. Liposomes

The discovery of liposomes originated from fundamental studies on phospholipid self-assembly conducted in the early 1960s by Alec D. Bangham and colleagues at the Babraham Institute in Cambridge, UK. While investigating the permeability properties of phospholipid membranes as models for biological systems, Bangham and Horne observed that when dry phosphatidylcholine films were hydrated in aqueous electrolyte solutions, the lipids spontaneously organized into closed, multilamellar vesicular structures. These structures, visualized by negative-stain transmission electron microscopy, exhibited alternating electron-dense and electron-lucent layers corresponding to lipid bilayers and aqueous compartments, respectively [20]. In subsequent work, they demonstrated that these bilayer assemblies could entrap ions and small solutes, thereby functioning as semi-permeable membranes that mimicked key physicochemical properties of the cellular plasma membrane [21]. The conceptual impact of these findings demonstrated that first, liposomes became indispensable model systems for biophysical membrane studies, and second, their ability to encapsulate hydrophilic and lipophilic molecules led to their exploration as drug-delivery systems. Subsequent methodological advances during the 1970s and 1980s, including sonication, extrusion, freeze–thaw cycling, and reverse-phase evaporation, enabled the production of liposomes with controlled size, lamellarity, and encapsulation efficiency, establishing the technological foundation for modern liposomal therapeutics [22].

Based on the number of lipid bilayers and diameter, liposomes are generally divided into three main categories [23,24]: Small Unilamellar Vesicles (SUVs) (20–100 nm) consist of a single phospholipid bilayer enclosing an aqueous core, normally produced by sonication or extrusion, and are used for applications requiring high circulation stability and fast tissue penetration. Large Unilamellar Vesicles (LUVs) (100–1000 nm) consist of a single bilayer but with a larger internal volume, allowing for higher encapsulation of hydrophilic drugs, and are often obtained through reverse-phase evaporation or ethanol injection techniques. Multilamellar Vesicles (MLVs) (500–5000 nm), composed of multiple concentric bilayers resembling an onion-like structure, easily formed by simple hydration of lipid films, exhibit higher stability but lower encapsulation efficiency and slower drug release.

Liposomes can also be categorized according to their lipid composition and surface functionalization, which affect their biological fate and targeting properties [25]. In this case we can find conventional liposomes which are composed mainly of natural or synthetic phospholipids and cholesterol susceptible to rapid clearance by the reticuloendothelial system (RES); stealth (PEGylated) liposomes, which incorporate polyethylene glycol (PEG)-conjugated lipids to form a hydrophilic corona that reduces opsonization and prolongs circulation time (e.g., Doxil^®^) [22]; cationic liposomes, which contain positively charged lipids such as DOTAP or DOTMA and are suitable for gene delivery due to electrostatic interaction with nucleic acids [26]; anionic or neutral liposomes which are for improved biocompatibility and reduced cytotoxicity; immunoliposomes which are functionalized with antibodies or ligands to enable targeted drug delivery to specific cell receptors or tissues (e.g., HER2-targeted liposomes in oncology) [27].

**Figure 2:**
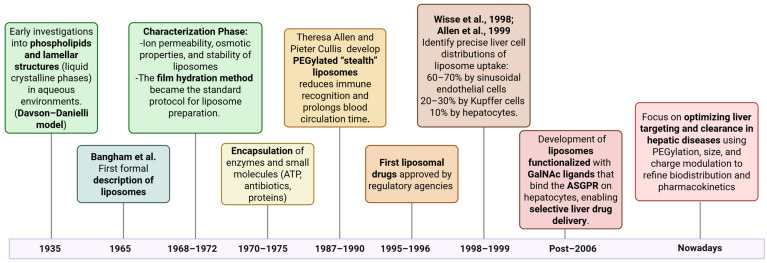
Timeline of Liposome research. The references covering the above timeline are as follows: [1,2,4,5,18,20,21,22,28].

An overview of the key research topics that have evolved during the last years is presented in Figure 2.

### 2.3. Extracellular Vesicles (EVs)

The heterogeneity of EVs has contributed to the identification of multiple mechanisms underlying their biogenesis, which serves as the basis for their classification. EVs can be classified into three main categories: Exosomes, microvesicles, and apoptotic bodies. Exosomes are small vesicles, generally less than 150–200 nm in diameter. They are formed intracellularly in specialized compartments known as multivesicular bodies (MVBs). When these MVBs fuse with the plasma membrane, their contents, including the intraluminal vesicles, are released into the extracellular space as exosomes [29]. On the other hand, microvesicles also known as ectosomes range from 50 to 10,000 nm and are produced by outward budding and shedding from the plasma membrane [12]. Apoptotic bodies range from 50 to 5000 nm in size and are released during apoptosis [12].

Exosomes are formed by the invagination of the endosomal membrane, leading to the formation of MVBs [29]. The biogenesis of these vesicle subtypes depends on multiple pathways intertwined and either dependent or independent of the endosomal sorting complex required for transport (ESCRT) pathways [30]. The ESCRT dependent pathway involves the interaction of ESCRT protein complexes (ESCRT-0, I, II, and III) including ALIX, VPS4, and TSG101 in a sequential manner to form the vesicles [30]. On the other hand, the ESCRT independent pathway includes lipid-dependent mechanisms including lipid rafts, and Tetraspanin-enriched microdomains (TEMs) abundant in CD81, CD9, and CD63 which regulate biogenesis and cargo sorting/packaging into exosomes [31]. Recently, a new role of the endoplasmic reticulum has come to light in the process of exosome release. The endoplasmic reticulum, together with small Rab GTPases like RAB27a/b, RAB7, RAB11, and RAB35, regulates MVB movement and fusion with the plasma membrane to enable exosome release [32]. Other proteins involved in exosome release include members of the SNARE proteins (YKT6, Vamp7), phospholipase D2, syndecan heparan sulfate proteoglycans, syntenin, and ADP ribosylation factor 6 (ARF6) [33].

**Figure 3:**
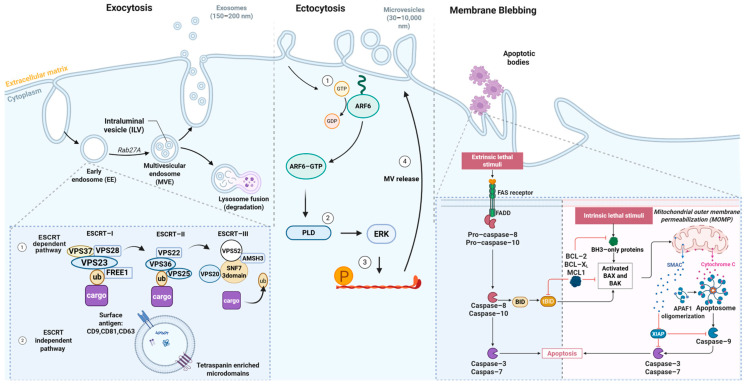
Biogenesis of Extracellular Vesicles.

Microvesicles (MVs) were initially identified as lipid-rich vesicles shed from platelet granules [9]. Unlike exosomes, MVs form by direct outward protrusion of the plasma membrane, involving calcium-dependent enzymes like scramblases and calpain that rearrange membrane lipids and proteins to facilitate MV formation and release [34]. However, their biogenesis is not heavily influenced by endosomal trafficking pathways as exosomes [12,24,35] (Figure 3).

The biogenesis of MVs involves actin filament remodeling, which produces the mechanical forces essential for their detachment from the plasma membrane. This process is regulated by small GTPases which control actomyosin contraction at the budding MV neck [36]. ARF6, a small Ras GTPase, regulates MV shedding through activation of phospholipase D, leading to the recruitment of ERK (extracellular signal-regulated kinase) to the plasma membrane. ERK activation initiates myosin light-chain kinase, resulting in phosphorylation of the myosin light chain, which facilitates actomyosin contraction and supports membrane fission, allowing for MV release [36].

Apart from exosomes and MVs, other types of vesicles include apoptotic bodies and the more recently identified exomeres. Apoptotic bodies are shed by cells undergoing the final stages of apoptosis. They are very heterogeneous in size and like other EV types carry biological cargo reflecting cells of origin such as histones, DNA fragments, and glycol-epitopes [37]. Although the mechanism of apoptotic body formation has not been fully established; current research indicates that a process known as apoptotic cell disassembly, involving several regulated morphological stages, may play a role [38].

Exomeres and supermeres are a novel subtype of EVs that were discovered recently. Smaller than 50 nm in diameter, they have been identified as additional non-vesicular nanoparticles. Exomeres have been shown to have distinct proteomic profiles compared to exosomes, whereas supermeres have been reported to be enriched with RNAs and show enhanced accumulation in tissues compared to exomeres and small EVs [39].

**Figure 4:**
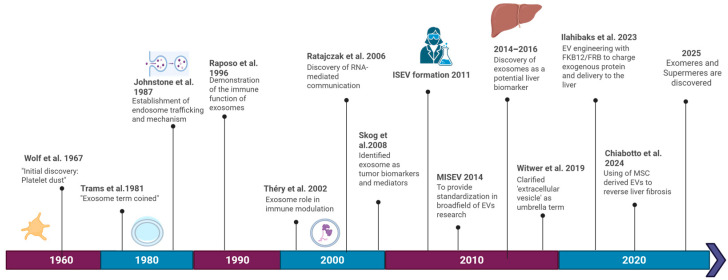
Timeline of Extracellular Vesicles research. The references covering the above timeline are as follows: [9,10,11,39,40,41,42,43,44,45,46,47].

Figure 4 provides an overview of the principal research areas that have emerged over the past 60 years.

### 2.4. Lipid Nanoparticles (LNPs)

LNPs are nanoscale delivery vehicles composed of lipid-based materials designed to transport therapeutic drugs or genetic cargo within the body. Their characteristic phospholipid-based structure enables the encapsulation of molecules either in the aqueous core or within the lipid layers, allowing for versatile payload loading [48].

**Figure 5:**
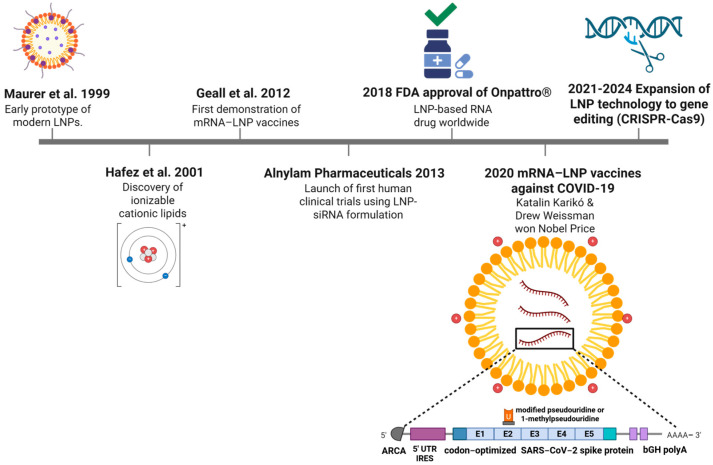
Timeline of LNP research. The references covering the above timeline are as follows: [50,51,52,53,54,55].

Beyond small-molecule drug delivery, LNPs have become indispensable in the field of nucleic acid therapeutics, particularly for the delivery of mRNA, siRNA, and gene-editing components. Their ability to protect nucleic acids from enzymatic degradation, facilitate cellular uptake, and promote endosomal escape has enabled significant advances in gene therapy, cancer immunotherapy, and, most notably, the rapid development and global deployment of mRNA vaccines during the COVID-19 pandemic [49]. These achievements have established LNPs as a versatile and clinically validated platform for precision molecular delivery. A summary of the major milestones in this field is provided in Figure 5.

This structural design supports controlled and targeted release, improving therapeutic effectiveness while minimizing off-target toxicity. LNPs also exhibit low inherent toxicity, making them well-suited for medical use [56]. The structure and composition of LNPs vary depending on the types of lipids used and the synthesis methods applied. Solid Lipid Nanoparticles (SLNs), for example, are formed from lipids that remain solid at room temperature [57]. SLNs can adopt one of three structural arrangements depending on how the drug is incorporated: a drug-enriched shell, a drug-enriched core, or a homogenous matrix [58]. Nanostructured Lipid Carriers (NLCs) represent a later generation of lipid nanoparticles developed to address limitations related to lipid crystallinity and polymorphic transitions in SLNs [59]. NLCs are produced using a mixture of solid and liquid lipids, resulting in a less ordered internal structure that enhances drug loading capacity and stability [59]. The arrangement of lipids and encapsulated compounds within these systems can vary, giving rise to distinct structural models [58]. Lipid–drug conjugates (LDCs) incorporate therapeutic molecules that are chemically linked to lipid moieties, improving solubility and delivery efficiency [60]. Polymer–Lipid Hybrid Nanoparticles (PLNs), in contrast, combine a polymeric core with a surrounding lipid layer, merging the stability and controlled release characteristics of polymers with the biocompatibility and membrane-like properties of lipids [61]. Over the past five years, numerous strategies have been explored to enhance the design and performance of solid lipid nanoparticles (SLNs). A recent review of patents and research trends highlighted progress in optimizing SLN formulations for therapeutic, cosmetic, and nutraceutical applications [62]. One notable advancement is the application of the Quality by Design framework, which improves the manufacturing of lipid nanoparticles and nano-emulsions by systematically controlling process variables to ensure product quality and safety [63]. Similarly, mathematical modeling and statistical optimization techniques have been employed to identify and regulate key formulation parameters in lipid-based nano-systems, including SLNs, NLCs, and nano-emulsions [64]. These methods provide consistent product performance by enabling precise control over factors such as lipid composition, surfactant concentration, and homogenization pressure [49].

More recently, researchers have focused on hybrid delivery systems that combine LNPs with biological membranes. For example, liquid crystalline lipid nanoparticles have been cloaked with cancer cell–platelet hybrid membranes to prolong circulation time and improve tumor targeting, thereby integrating the functional advantages of both membrane types [65]. Importantly, these capabilities have positioned LNPs as promising tools for treating genetic liver diseases by enabling the delivery of gene-editing components or RNA-based therapies directly to hepatocytes.

## 3. Cargo Incorporation and Liver-Specific Targeting Using Nanovesicles

### 3.1. General Mechanisms of Loading

Nanovesicles can encapsulate or associate a wide range of therapeutic cargoes, and several general loading strategies have been developed to optimize their efficiency and stability. Passive loading relies on the natural diffusion or spontaneous partitioning of molecules into or across the vesicle membrane and is particularly suitable for hydrophobic compounds that readily integrate into lipid bilayers. In contrast, active loading strategies employ external drivers, such as pH gradients, ion gradients, or membrane permeabilization, to promote the selective accumulation of hydrophilic drugs or nucleic acids within the vesicle lumen. Additionally, surface modification approaches, including chemical conjugation, electrostatic binding, or ligand–receptor interactions, can be used to anchor therapeutic macromolecules to the outer vesicle surface while preserving internal volume for other cargoes. The choice of loading method depends on the physicochemical properties of the cargo, the desired release profile, and considerations related to vesicle integrity and biological compatibility. Collectively, these strategies allow nanovesicles to function as versatile and tunable carriers for diverse bioactive molecules, supporting their development for targeted therapeutic delivery.

**Figure 6:**
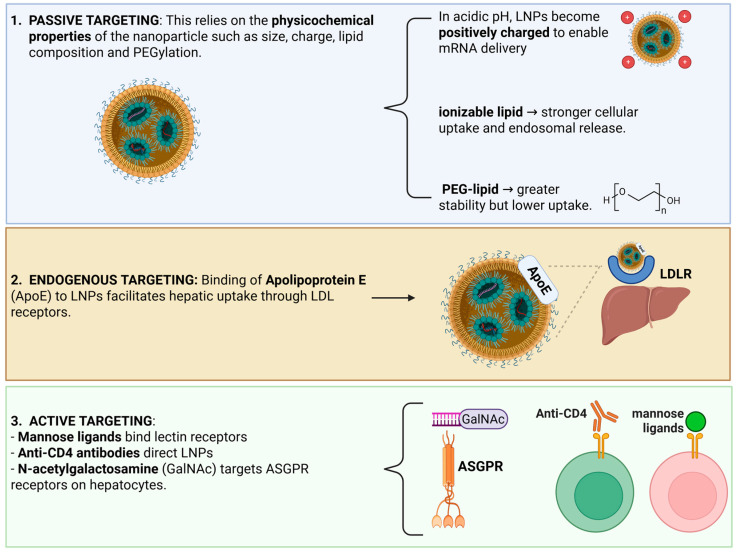
Overview of targeting strategies used to enhance delivery to the liver.

**Figure 7:**
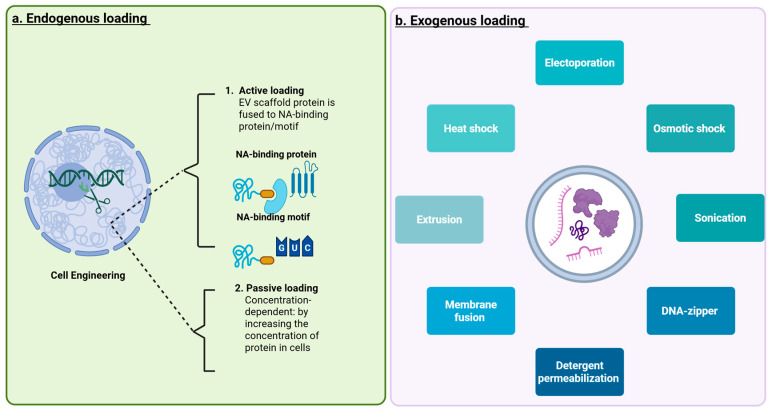
Overview of extracellular vesicle (EV) cargo loading approaches. (**a**) Endogenous loading occurs during vesicle formation within the parent cell. (**b**) Exogenous loading applied to EVs after their isolation.

**Figure 8:**
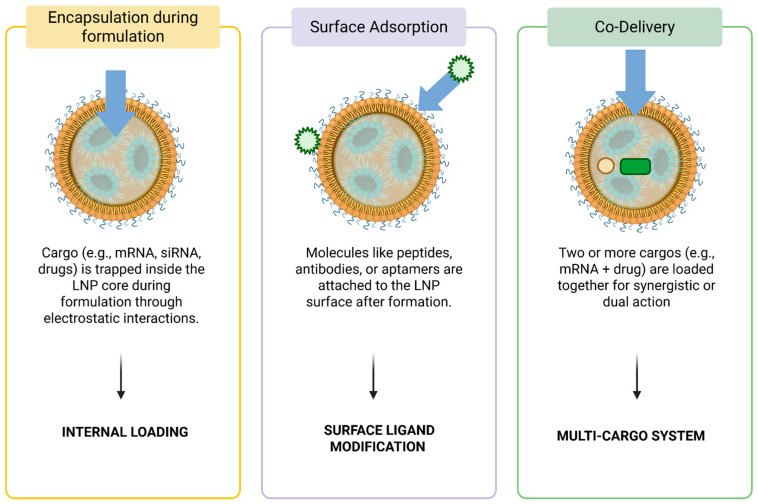
General mechanisms for cargo loading into lipid nanoparticles (LNPs).

The choice of loading strategy is influenced by the structural and functional characteristics of the nanovesicle used, and an overview of these approaches is provided in Figure 6, Figure 7 and Figure 8.

### 3.2. General Strategies for Targeting Nanovesicles

Targeting strategies for nanovesicles are designed to enhance the selective delivery of therapeutic cargo to specific tissues or cell populations while minimizing off-target effects. Passive targeting exploits the natural biodistribution and physicochemical properties of nanovesicles, such as size and membrane composition, which can favor accumulation in organs like the liver or spleen. In contrast, active targeting involves modifying the vesicle surface with specific ligands, such as antibodies, peptides, aptamers, or small molecules, that recognize and bind receptors overexpressed on target cells, thereby improving cellular uptake and localization. Additionally, membrane engineering approaches, including lipid rearrangement or incorporation of targeting moieties during vesicle formation, can further refine tissue specificity and circulation profiles. Some nanovesicles, such as extracellular vesicles, also possess intrinsic homing properties derived from their parent cells, which can be leveraged to achieve organ- or cell-specific delivery without extensive modification. Collectively, these targeting strategies enable nanovesicles to function as precise delivery platforms, supporting the development of more effective and tailored therapeutic interventions.

Figure 6 illustrates the different strategies used to modify targeting, highlighting key liver-specific molecules that can facilitate hepatic delivery.

The physicochemical properties of nanovesicles strongly influence their fate in vivo. Following systemic administration, nanovesicles interact with various bioactive proteins in the bloodstream, including immunoglobulins, lipoproteins, complement proteins, apolipoproteins, and albumin, leading to the formation of a protein corona [66]. Receptors for certain protein corona components, such as apolipoproteins and lipoproteins, are expressed on hepatocytes, facilitating nanovesicle uptake by the liver. In addition to the protein corona, factors such as size, stiffness, shape, and surface modifications also affect the biodistribution of nanovesicles in vivo [66].

Surface modifications are designed to enhance or prevent interactions between nanovesicles and cells. For example, polyethylene glycol (PEG) is commonly used to modify nanovesicle surfaces and reduce rapid clearance from the bloodstream. PEGylation increases the hydrophilicity of nanovesicles, minimizing cellular uptake and thereby prolonging their circulation half-life. In addition, PEGylation helps prevent recognition and interaction with host antibodies and enzymes, improving nanovesicle stability in vivo [66]. Beyond PEGylation, certain proteins such as CD47, known for delivering a “don’t eat me” signal that suppresses immune cell phagocytosis, can also be incorporated on the nanovesicle surface. For instance, nanovesicles coated with biomimetic red blood cell membranes exploit surface-expressed CD47 to prolong circulation time and decrease clearance rates from the bloodstream [67].

### 3.3. Selective Liver Tropism in EV-Mediated Delivery

Cargo can be loaded into EVs following two main routes: Endogenously or exogenously (Figure 7). The endogenous route involves either passive cargo introduction into cells, or the cell’s EV biogenesis machinery (Figure 2) to load cargo into vesicles prior to release. The latter involves genetic engineering, loading genetic substances into EVs. This approach requires cells to be genetically engineered to express EV scaffold proteins fused with a protein or RNA of interest, which is subsequently incorporated into the exosomes as they form. While this method is effective for loading specific proteins or RNA molecules, it necessitates the use of complex molecular biology techniques and may not be suitable for all types of therapeutic agents [68].

In contrast, exogenous cargo loading refers to techniques applied after EVs have been isolated from cells. Physical and chemical methods such as electroporation, sonication, freeze–thaw cycles, extrusion, or co-incubation are used to facilitate the entry of therapeutic molecules into pre-formed vesicles [69]. These methods allow researchers to introduce a wide range of therapeutic cargos including siRNA, mRNA, proteins, and small molecule anticancer drugs like doxorubicin or sorafenib into the exosomal lumen or membrane [15,69,70] (Figure 7).

Engineered EV cargo strategies for liver therapy emphasize deliberate control of both what is loaded and how it is routed into vesicles. Endogenous loading (Figure 7a) by genetically engineering the producer cell is widely used for RNAs and proteins. Regarding RNAs, producer cells are transfected or viral transduced to overexpress specific miRNAs/siRNAs/lncRNAs or full-length mRNAs, yielding EVs naturally enriched in the desired nucleic acids. Examples of therapeutic miRNA cargos for liver fibrosis include miR-486-5p and miR-122 [71,72]. For protein therapeutics, exogenous loading methods are most employed. In particular, fusion-protein strategies are widely used to bias sorting target proteins into EVs. In these approaches, the protein cargo is genetically fused to canonical exosomal scaffold or tetraspanin proteins, e.g., N-terminal fusions to CD63 or CD81), to endosomal/lysosomal membrane proteins such as Lamp2b, or to budding-associated adaptors like ARRDC1. Alternatively, fusion to lipid-anchor motifs can promote association with the EV membrane. These strategies increase the efficiency of partitioning the therapeutic protein into either the EV lumen or membrane and have been used to harness pathways such as the Lamp2a-mediated loading of KFERQ-motif proteins into exosomes [73,74]. Building on these principles, the method provides direct evidence for functional in vivo protein delivery. In this system, Cre recombinase displayed on engineered EVs successfully mediates recombination in reporter hepatocytes following systemic administration, demonstrating that EV-based protein therapeutics can achieve targeted activity in liver tissue [46]. Together, these approaches highlight how rational fusion design and EV surface engineering can enable efficient, physiologically relevant delivery of therapeutic proteins.

Regarding small molecules and hydrophobic drugs such as siRNA and oligonucleotide, exogenous post-isolation loading (Figure 7b) remains the method of choice. Representative methods include sonication/extrusion and membrane-fusion-mediated incorporation [75,76]. Hybrid approaches such as pre-complexing oligonucleotides with cationic lipids or nanoparticles and then cloaking them with EV membranes, combine the stability of synthetic carriers and the biocompatibility/tropism of EVs [76]. Surface engineering for active targeting, for example, display of GalNAc ligands or peptide ligands using RNA-nanotechnology or Lamp2b/CD63 fusions for receptor-mediated uptake has been demonstrated to redirect EV tropism to hepatocytes or hepatocellular carcinoma (HCC) cells [77]. Finally, biological sorting pathways such as ESCRT components, tetraspanin-enriched microdomains, and chaperone-mediated motifs (KFERQ/LAMP2A) are now exploited to design endogenous loading signals that selectively package therapeutic proteins or RNAs into EV subpopulations [74,75].

Turning to targeting strategies, engineered EVs can be customized to selectively reach specific liver cell populations, such as hepatocytes, hepatic stellate cells (HSCs), Kupffer cells, and liver sinusoidal endothelial cells (LSECs), depending on the therapeutic applications. Several experimental studies have shown the feasibility of directing engineered EVs to hepatocytes using receptor-ligand interactions. For instance, Lamp2b-fused exosomes displaying a galactose or GalNAc peptide efficiently target the asialoglycoprotein receptor (ASGPR) on hepatocytes, leading to enhanced accumulation in liver parenchyma and improved gene transfer efficiency [78]. In vivo biodistribution studies have confirmed that engineered EVs can selectively deliver active proteins such as Cre recombinase to hepatocytes, validating their potential as gene delivery vehicles [46]. Another important target population is the hepatic stellate cell, the major driver of fibrogenesis. EVs engineered to overexpress antifibrotic miRNAs, such as miR-486-5p or miR-378c, or to carry proteins that suppress TGF-β and collagen pathways, have shown strong antifibrotic effects in experimental models of chronic liver injury and non-alcoholic steatohepatitis [79], Table 1. In parallel, engineered EVs are being developed for the treatment of HCC through the delivery of tumor-suppressive RNAs or chemotherapeutic agents. For example, GalNAc-displaying exosomes loaded with miR-122 or paclitaxel selectively accumulated in HCC cells and suppressed tumor proliferation both in vitro and in vivo [77]. The combination of active ligand targeting with controlled cargo release offers an effective strategy to overcome the limitations of conventional drug carriers in liver cancer therapy. Recent reviews further highlight the emergence of hybrid and synthetic-bio EV platforms that integrate genetic engineering, chemical conjugation, and nanomaterial hybridization to achieve unprecedented precision in liver targeting [75,76].

Kupffer cells and macrophages also represent key targets for immunomodulatory EV therapy. Engineered MSC-derived EVs loaded with anti-inflammatory cytokines or miRNAs have been shown to polarize macrophages toward an M2 phenotype, thereby reducing hepatic inflammation in models of toxin-induced and metabolic liver damage [80]. Likewise, EVs designed to express ligands for macrophage surface receptors can exploit the natural phagocytic properties of Kupffer cells to achieve high local concentrations of therapeutic agents in the liver. At the vascular interface, engineered EVs have been explored to protect or regenerate LSECs, which play a key role in hepatic microcirculation and oxidative stress responses.

Moreover, LSECs are increasingly recognized as central regulators of intravascular waste clearance. As outlined in the comprehensive review by Bhandari [81], LSECs function as the liver’s professional pinocytes, equipped with high-affinity scavenger receptors and an extensive endocytic vesicular system that enables the rapid removal of soluble macromolecules and small nanoparticles from the bloodstream. Bhandari and colleagues emphasize that a wide range of endogenous waste ligands including modified proteins, lipoprotein remnants, extracellular matrix fragments, and diverse classes of nanomaterials are cleared mainly by LSECs, with negligible uptake in Kupffer cells, reinforcing a longstanding functional specialization within the sinusoidal niche. This view is strongly aligned with the classical dual-cell principle of hepatic clearance, originally formalized by Sorensen et al. [82]. In this framework, LSECs are described as high-capacity endocytic cells optimized for the uptake and catabolism of small soluble substrates and sub 200 nm particulate material through clathrin mediated endocytosis, whereas Kupffer cells serve as professional phagocytes responsible for the removal of larger particles, cellular debris, and pathogens. Early foundational studies by Smedsrod and colleagues [83] further established the role of scavenger endothelial cells as a dedicated intravascular clearance system with exceptional ability to eliminate circulating macromolecules such as hyaluronan, challenging the earlier assumption that hepatic macrophages alone constituted the core of the reticuloendothelial system.

Historically, a quantitative ultrastructural description of LSEC fenestrations has relied on electron microscopy. However, Monkemoller et al. [84] introduced a major advance by applying direct stochastic optical reconstruction microscopy (dSTORM) to visualize LSEC fenestrations at near–electron-microscopy resolution under optical conditions. Using this approach, individual fenestrations were resolved with a localization precision of ~20 nm, enabling accurate measurement of pore size distributions in intact plasma membranes. The analysis revealed that fenestrations are not confined to a narrow size range but rather display a broad nanoscale distribution. While the mean fenestration diameter was ~120 nm (119.8 ± 37.6 nm), super-resolution imaging demonstrated the coexistence of very small fenestrations (<50 nm) together with larger pores extending up to approximately 180–200 nm. Importantly, these larger fenestrations constitute a substantial fraction of the total population, indicating that LSEC sieve plates consist of a heterogeneous ensemble of pores spanning nearly one order of magnitude in size, rather than a uniform array of ~50 nm openings [84].

Together, these works support a well-established model in which LSECs, rather than Kupffer cells, represent the dominant hepatic cell type responsible for clearing small particles and soluble macromolecules via receptor-mediated endocytosis. However, despite this strong conceptual framework, there remains limited discussion and relatively sparse experimental investigation into the full breadth of LSEC-mediated clearance, particularly in the context of nanomedicine, liver disease, and complex particle architectures. As highlighted by Bhandari et al. [81], significant knowledge gaps persist regarding how LSEC clearance capacity is modulated under physiological versus pathological conditions, indicating that this field while conceptually robust remains moderately explored [81].

### 3.4. Selective Liver Tropism in LNP-Mediated Delivery

LNPs can incorporate a broad spectrum of therapeutic molecules, and their cargo loading strategies are typically tailored to the physicochemical properties of the payload. Hydrophobic small molecules can be readily embedded within the lipid bilayer through passive incorporation, leveraging their affinity for the lipid environment. In contrast, hydrophilic or charged biomolecules, such as nucleic acids, generally require active loading strategies driven by controlled electrostatic interactions; a positively charged lipid component facilitates complexation with negatively charged nucleic acids, enabling efficient encapsulation within the nanoparticle core. Additionally, microfluidic mixing and formulation parameters influence particle size, membrane fluidity, and encapsulation efficiency, allowing fine-tuning of stability and release kinetics. Beyond internal encapsulation, surface modification approaches, including ligand conjugation or polymer coating, can enable co-loading of additional molecules or support targeted delivery to specific tissues [85]. Together, these versatile loading strategies enable LNPs to act as adaptable carriers for diverse bioactive agents, facilitating their growing use in therapeutic delivery (Figure 8).

LNPs are well known for their preferential accumulation in the liver, a feature that has been widely leveraged for treating hepatic diseases. This liver tropism arises from both the physiological characteristics of the liver and the interaction of LNPs with circulating apolipoprotein E (ApoE) [86,87]. Upon systemic administration, LNPs rapidly bind ApoE, which mediates their uptake into hepatocytes primarily through low-density lipoprotein (LDL) receptors serving as a form of endogenous liver targeting [88]. This mechanism underlies the delivery pathway of Patisiran, the first FDA-approved siRNA-based drug for treating polyneuropathy associated with hereditary transthyretin-mediated amyloidosis (hATTR). By exploiting this route, Patisiran effectively targets the liver to reduce the production of the transthyretin (TTR) protein, achieving its therapeutic effect [54].

However, it is essential to differentiate biodistribution from functional delivery Although LNPs may accumulate efficiently in the liver, this does not guarantee productive intracellular delivery or therapeutic efficacy. For instance, Sato et al. demonstrated that hepatocyte uptake and intracellular trafficking differ depending on the ionizable lipid structure, and that the pKa of these lipids influences intrahepatic LNP distribution, highlighting how chemical modifications can modulate functional delivery [89]. Similar trends are seen in LNP-based siRNA therapies, where gene silencing outcomes depend on factors such as lipid composition, dose, cell-type interactions, and, critically, endosomal escape efficiency [90].

Advances in selective organ targeting nanoparticles further demonstrate that manipulation of the lipid compositions can shift biodistribution across the liver, spleen, and lungs [91]. For liver-selective delivery, the incorporation of ionizable cationic lipids, such as DODAP, increases hepatic accumulation through enhanced serum protein adsorption, including ApoE, which directs LNPs to hepatocytes via LDL receptors or alternative receptor-mediated pathways depending on the recruited protein corona. Investigation into optimizing liver-targeted LNPs has identified that specific ionizable lipids, such as MC3 and cKK-E12, possess pKa values tuned for hepatocyte uptake coupled with improved endosomal escape capabilities [92]. Helper lipids further modulate biodistribution and cellular routing, with DOPE enhancing ApoE-mediated liver uptake, whereas DSPC biases distribution toward the spleen [93]. Additional modifications, including the incorporation of cationic cholesterol, further broaden delivery to extrahepatic tissues, such as the lung and heart [94]. Surface chemistry confers a further role: PEGylation enhances circulation stability, but cleavable PEG variants promote efficient hepatic release, while ligand-modified PEG moieties, such as mannose-PEG, allow for receptor-specific targeting [93].

Ultimately, precise cellular targeting within the liver is necessary to achieve therapeutic efficacy. Specific targeting of nonparenchymal cells like hepatic stellate cells and macrophages resulted in reduced markers of fibrosis and specific restoration of liver function, thus promoting tissue repair and fibrosis reversal [95,96,97]. In a similar vein, LNPs encoding HGF/EGF or NM-FGF19 mRNA showed therapeutic efficacy in models of chronic liver injury and metabolic dysfunction via selective activation of the respective hepatic cell populations [98].

Other antitumor agents such as siRNA, miRNA, and mRNA have also been delivered as therapeutic cargo using delivery systems in preclinical models of HCC, revealing promising safety, tolerability, and efficacy results. For example, Tabernero et al., in a phase 1 clinical trial, successfully administered LNPs (ALN-VSP) encapsulated with siRNAs that targeted kinesin spindle protein and vascular endothelial growth factor to treat patients with liver metastases, leading to tumor regression in almost 50% of the patients in that trial [53]. Since then, various groups have attempted to utilize RNAi platforms in conjunction with chemotherapeutic agents to target liver cancer [99,100,101,102,103].

Another approach is to redesign the lipid composition of LNPs to allow for controlled release of chemotherapeutic agents in response to specific stimuli, such as pH responsive [104], temperature sensitive [105], photo sensitive, magnetic-sensitive, or ultrasound-guided lipids [86]. For instance, Li et al. showed that the inclusion of cationic lipids in the LNP bilayer induced direct internalization into tumor cells through a conformational change triggered by the acidic tumor microenvironment. This strategy increased the targeting of the tumor specifically while decreasing the toxicity of the drug and off-target effects on normal hepatocytes [104].

In parallel, GalNAc-conjugated LNPs enable hepatocyte-specific uptake independent of the LDL receptor via the asialoglycoprotein receptor [55,106]. He et al. exploited this possibility for the design of nanovesicles by incorporating GalNAc-modified cell membranes into biodegradable mesoporous LNPs, resulting in an efficient and safe siRNA delivery system targeting hepatocytes with effective silencing of PCSK9 gene expression for the treatment of non-alcoholic fatty liver disease [107].

Together, these studies illustrate how rational design of LNP composition, surface chemistry, and protein corona interactions can finely tune liver tropism, intrahepatic distribution, and functional therapeutic cargo delivery.

Gene therapy attempts to correct or modulate genetic information inside the cells in order to treat diseases at its molecular origin. A central challenge in its application is the safe and efficient delivery of nucleic acids, such as mRNA, siRNA, or DNA, to specific tissues without inducing toxicity or immune activation. LNPs have emerged as a leading platform for nucleic acid delivery because they encapsulate and protect genetic cargo, enable efficient cellular uptake, and can be engineered for organ-selective biodistribution. In several preclinical metabolic disease models, LNP-formulated mRNA has demonstrated therapeutic potential. For example, An et al. evaluated meth-oxy-polyethylene glycol lipid nanoparticles (Mtx-LNPs) carrying hMUT mRNA in two mouse models of methylmalonic acidemia (MMA), showing increased hepatic MUT activity, improved growth and survival, and reduced toxic metabolite accumulation [108]. More recently, Cao et al. used Mtx-LNPs to deliver a codon-optimized G6PC mRNA variant (S298C) in a G6Pase-null mouse model of glycogen storage disease type Ia, resulting in: a dose-dependent improvements in fasting glucose levels, reduced hepatic steatosis, and normalization of biochemical markers [109]. Similarly, Yamazaki et al. demonstrated that intravenous administration of LNP-encapsulated human OTC mRNA restored urea cycle function in a mouse model of ornithine transcarbamylase deficiency, leading to hepatic expression of functional hOTC protein. Collectively, these studies highlight the promise of LNP-mediated mRNA delivery for treating inherited metabolic liver disorders, supporting ongoing clinical development in this field [110], Table 1.

### 3.5. Selective Liver Tropism in Liposomes-Mediated Delivery

As a drug delivery platform, liposomes offer advantageous drug-loading capacity and inherent targeting properties. In the context of acute liver failure, these liposomal carriers can be engineered to selectively accumulate in injured hepatocytes, thereby enhancing therapeutic concentration at the site of damage and reducing systemic exposure [111]. In particular, liposomes can encapsulate and deliver agents with anti-inflammatory, antioxidant, and cytoprotective activities, which help mitigate oxidative stress, suppress excessive inflammatory responses, and protect hepatocyte integrity in acute liver injury [111,112].

**Figure 9:**
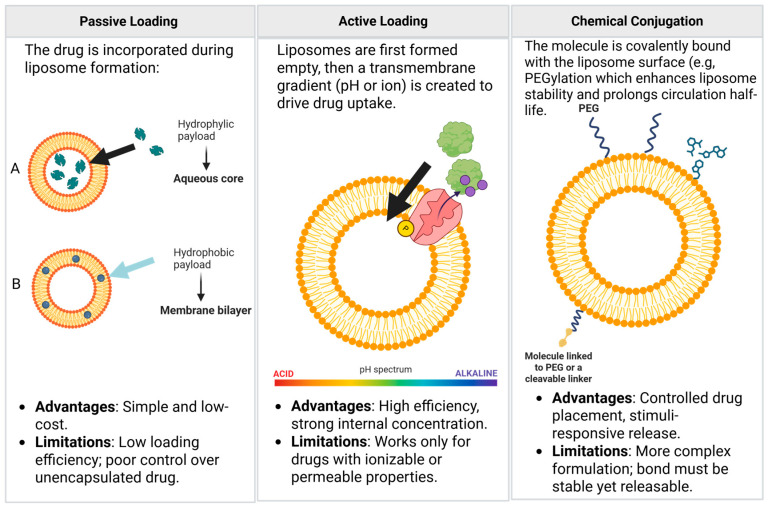
General mechanisms for cargo loading into Liposomes. The black arrows in passive and active loading depict internalization of cargo. The blue arrow (passive loading B), depicts attachment of cargo on membrane bilayer.

Efficient loading of therapeutic agents into liposomes is essential to achieve optimal drug encapsulation, stability, and controlled release. Two major strategies are used: passive and active loading (Figure 9).

The mechanism of passive loading depends entirely on the drug’s solubility. Hydrophilic drugs which are dissolved in the aqueous phase that forms the core of the liposome where a fraction of the drug is mechanically entrapped within the internal aqueous volume of the liposome upon vesicle closure [113,114]. In this case the encapsulation efficiency (EE) is generally low (often 10–30%) as it is directly proportional to the encapsulated aqueous volume and the drug concentration in the bulk solution [113,114]. Lipophilic drugs on the other hand, are dissolved along with the phospholipids in the organic solvent prior to film formation. During the self-assembly process, the drug molecules intercalate and partition into the hydrophobic region of the lipid bilayer [114]. In this case the encapsulation efficiency is higher due to their strong affinity for the lipid bilayer, making passive loading the preferred method for this class of compounds [113,114] (Figure 9).

In general passive loading methods are simple and scalable, are suitable for drugs of any polarity but they have a low encapsulation efficiency for hydrophilic drugs (typically 10–30%) and passive incorporation of certain lipophilic drugs can sometimes lead to bilayer destabilization or rapid drug leakage [113].

Active Loading, commonly known as remote loading, is a post-formulation drug loading technique used to achieve high EE and superior retention of water-soluble and amphiphilic drugs. Unlike passive methods, it does not rely on the trapped aqueous volume but instead utilizes a transmembrane gradient established after liposome formation [115]. The pH gradient method is the gold standard for remote loading, particularly successful for amphiphilic weak bases (e.g., Doxorubicin, the drug in Doxil^®^) [115,116]. It starts with a gradient creation in which liposomes are prepared with an acidic internal buffer and the external medium is exchanged for a neutral or slightly alkaline buffer. This establishes a significant transmembrane pH gradient. Then follows the drug permeation phase where the weak base drug exists in equilibrium between its non-ionized form and its ionized form [115].

The liver is a natural target for many nanovesicles because of its fenestrated sinusoidal endothelium, resident phagocytic Kupffer cells, and abundant lipoprotein receptors. This high baseline uptake can be an advantage or an off-target sequestration of particles intended for other organs, so rational design must exploit liver biology or avoid it depending on the therapeutic goal [117,118].

For example, hepatocytes are targeted when therapy needs to edit metabolic pathways, silence viral genes, or treat hepatocyte-derived tumors exploiting ASGPR and ApoE, mediated uptake [117,118]. Kupffer cells are targeted for immunomodulation in steatohepatitis, alcoholic liver disease, or to modulate fibrogenesis, mannose or other macrophage-directing ligands are used to bias uptake to these cells [119,120]; HSCs selectively targeted to deliver anti-fibrotic siRNA or drugs, vitamin A conjugation is a widely used strategy because quiescent and activated HSCs naturally uptake retinoids [121,122]; LSECs and dendritic cells targeting can be used for tolerogenic vaccination or immune modulation [123].

Clinical applications have demonstrated that passive hepatic accumulation of liposomes can be leveraged across multiple liver diseases, even in the absence of specific targeting ligands. In hepatocellular carcinoma (HCC), passive deposition driven by tumor-associated vascular permeability has enabled improved intratumoral drug delivery. The clinically approved PEGylated liposomal doxorubicin (Doxil^®^) showed enhanced retention in liver tumor models and reduced systemic toxicity compared to free doxorubicin [124]. Additional studies using arabinogalactan-conjugated liposomes delivering doxorubicin confirmed that, although ligand decoration can increase tumor selectivity, non-targeted liposomal formulations also achieve substantial drug accumulation within hepatic tumors due to the altered vascular architecture [125].

Passive hepatic targeting has similarly been exploited in viral hepatitis. For instance, liposome-encapsulated interferon-α achieved superior hepatic retention and prolonged antiviral activity in HBV and HCV animal models relative to free cytokine administration [126]. Liposomal lamivudine formulations have also been evaluated for HBV therapy, demonstrating preferential liver deposition, sustained release, and reduced systemic exposure while preserving antiviral potency [127]. Inflammatory liver diseases provide another clinically relevant context in which passive liposomal accumulation is therapeutically advantageous. In preclinical models of non-alcoholic steatohepatitis (NASH), liposomes carrying anti-inflammatory or anti-fibrotic agents such as curcumin and calcitriol displayed selective hepatic uptake and mitigated fibrosis progression by modulating local immune and stellate-cell activity [123]. Similar benefits have been observed in fibrotic and cirrhotic models, where plain liposomal formulations of silymarin or pirfenidone delivered antifibrotic effects by attenuating TGF-β signaling pathways directly within affected hepatic tissues [128].

Together, these clinical and preclinical findings underscore that, even without active ligand incorporation, passive liposome accumulation can be exploited as a practical therapeutic mechanism across diverse liver pathologies, including HCC, viral hepatitis, and inflammatory or fibrotic liver disease.

Active targeting of liposomes to the liver, the second method, refers to the intentional modification of liposomal surfaces with targeting ligands that bind selectively to receptors expressed on specific hepatic cell types, hepatocytes, Kupffer cells, HSCs, or LSECs. The most compelling use cases are those in which the therapeutic effect requires intracellular delivery to a specific population, for example, silencing profibrotic genes in hepatic stellate cells or delivering cytotoxics to hepatocellular carcinoma cells or where limiting exposure of non-target liver compartments would reduce toxicity. Active targeting therefore seeks to improve the therapeutic index, increasing efficacy in the target cell while reducing off-target effects in other hepatic or systemic cells [117,125].

Active targeting for HCC has focused on ASGPR (galactose/GalNAc), tumor antigen antibodies, and peptides against markers of aggressive tumor clones. Targeted liposomal chemotherapies often demonstrate superior cellular uptake, increased intratumoral retention and better tumor control in orthotopic models than untargeted liposomes. However, tumor heterogeneity and downregulation of the chosen receptor in subclones can limit efficacy, combination strategies often perform better [117,125,129].

For therapies that must enter hepatocytes, ASGPR-targeted liposomes improve hepatocyte specificity and reduce delivery to Kupffer cells. Reports of galactosylated liposomal formulations carrying antiviral drugs show enhanced hepatic delivery and improved pharmacodynamics in animal models [130,131].

In diseases driven by macrophage activation, like NASH/ALD, mannose or scavenger receptor-targeted liposomes successfully bias payloads toward Kupffer cells and pro-inflammatory monocyte-derived macrophages. Delivering anti-inflammatory small molecules or miRNA to this compartment reduces hepatocellular injury and collagen induction in preclinical NASH models [123,132], Table 1.

Together, these advancements highlight liposomes as a flexible platform capable of directing therapeutics to distinct hepatic cell populations based on surface ligand design. While continued optimization is needed to improve stability, dosing durability, and manufacturing scalability, the demonstrated ability of liposomes to reshape intrahepatic drug distribution provides a strong foundation for their integration into next-generation liver-directed therapies.

### 3.6. Viral Therapies as the Current Standard for the Treatment of Liver Diseases

Although viral vectors are not classified as nanovesicles, it is important to highlight their relevance, particularly adeno-associated viruses (AAV), which remain among the most efficient platforms for liver-targeted gene delivery. They provide a critical benchmark against which emerging nanovesicle-based technologies can be evaluated. AV serotypes such as AAV2, AAV8, and AAV9 exhibit strong intrinsic hepatotropism, which has been further enhanced through capsid engineering strategies, including directed evolution, rational mutagenesis, and peptide display. Seminal studies by Mingozzi et al. and Bell et al. defined the immunological and transduction features of modern hepatotropic AAV capsids [133,134]. Subsequent vector genome improvements using hepatocyte specific promoters, miRNA detargeting, and glycan binding motifs, have further enhanced liver selectivity [134]. This is underscored by several successful preclinical programs, including early work such as OTC gene-replacement study by Morizono et al. [135], in which a recombinant adenoviral vector expressing the human ornithine transcarbamylase (OTC) gene was delivered to OTC-deficient murine models [135]. The vector successfully restored hepatic OTC expression, reduced ammonia accumulation, and improved the metabolic crises characteristic of OTC deficiency. Although the effect was transient, partly due to the non-integrating nature of the vector, this study provided essential proof of concept that targeted hepatic gene transfer can correct inborn metabolic defects [135]. Similarly, Nathwani and colleagues phase I/II trial in hemophilia B demonstrated the clinical potential of AAV8-mediated liver gene delivery by a single intravenous infusion of an AAV8 vector expressing factor IX under a liver-specific promoter, resulting in sustained FIX expression for several years and a marked reduction or elimination of prophylactic clotting-factor infusions [136]. Despite these results the use of AAV vectors presented some significant safety critical issues: the fatal inflammatory response reported by Raper et al. and dose-related hepatotoxicity summarized by Duan et al. showed the need for process improvements to reduce reactogenic contaminants and to optimize dosing [137,138]. In fact even if vector production and purification methods have been improved to reduce the immunogenicity, a number of challenges persist, including high-dose requirements, complement activation, transient thrombocytopenia, episomal dilution of AAV genomes in proliferating pediatric hepatocytes and the presence of pre-existing anti-AAV antibodies which limits patient eligibility and requires immunosuppression to maintain long-term expression [139].

However, recent capsid innovations illustrate how vector engineering continues to evolve. Shen et al. [140] augmented hepatocyte entry through the introduction of glycan-binding motifs, mimicking natural hepatotropic viruses, whereas Jin et al. [141] extended circulation by means of albumin-binding peptide shields that utilize FcRn recycling. In parallel, lentiviral vectors (LVs) offer advantages in settings where stable genome integration is required. Work from Cantore et al. and Canepari et al. showed durable liver transduction using integrating LVs platforms that bypass pre-existing immunity and maintain expression throughout hepatocyte turnover [142,143]. Innovations such as CD47hi-LV from Milani et al. and metabolic-conditioning strategy from Canepari et al. dramatically increased in vivo liver transduction while reducing phagocytic clearance [143,144]. Furthermore, early work demonstrated that systemic administration of LVs triggers a type I interferon (IFN-α/β) response that restricts hepatocyte gene transfer and promotes vector clearance in vivo; in IFN-α/β receptor–deficient mice, transduction efficiency and stable transgene expression in hepatocytes were dramatically increased [145]. Moreover, Mates et al. [146] have developed hyperactive variants of the Sleeping Beauty (SB) transposase, including SB100X, by iterative molecular evolution. These optimized enzymes had much improved DNA-cutting and integration kinetics, enabled more efficient and stable genomic insertion when combined with an LV-delivered transposase system.

Hybrid viral–nonviral approaches now bridge the strengths of several platforms. Hybrid viral–non-viral approaches are also emerging: for instance, AAV vectors cloaked in exosome membranes (“AAVExo”) have been developed to enhance liver transduction and help bypass pre-existing neutralizing antibodies [147].

## 4. Translational and Clinical Potential of Nanovesicles in Liver Disease Therapy

The liver plays a central role in maintaining whole body metabolic homeostasis, including regulation of glucose, lipid, and amino acid metabolism, detoxification, and protein synthesis. As a result of these essential functions, it is also a primary site for the development of a range of disorders. These include HCC, various monogenic metabolic diseases, such as Wilson’s disease and familial hypercholesterolemia, as well as numerous other congenital and acquired hepatic conditions. Current treatment strategies can alleviate symptoms but remain insufficient, as a high risk of patient mortality remains [148]. Liver transplantation continues to be the most effective therapeutic option for correcting liver diseases. However, donor shortages and the inability of transplantation to reverse preexisting neurological damage highlight the urgent need for alternative therapeutic strategies [149].

Efforts to develop such alternatives began in the 1990s with viral vector-based gene therapy, which initially showed great promise. Nonetheless, safety concerns, particularly severe immune reactions, led to major setbacks, including life threatening inflammatory responses in adenoviral based liver gene therapy trials [137,150]. These adverse outcomes, which had not been predicted in preclinical studies, revealed critical flaws in trial design and resulted in a decade long stagnation in the field [150].

To address these limitations, researchers have explored non integrating approaches such as mRNA-based therapeutics. Unlike viral vectors, mRNA therapies eliminate the risk of genomic insertion, improving their safety profile. For example, Prieve et al. developed a hybrid mRNA technology (HMT) delivery system that combines an N-acetylgalactosamine (GalNAc)-targeted polymer micelle with a cationic lipid-based nanoparticle to deliver mRNA directly to hepatocytes and promote endosomal escape [151]. In mouse models of ornithine transcarbamylase deficiency (OTCD), this platform restored OTC enzyme synthesis and improved disease symptoms. Despite these encouraging results, the HMT system’s complexity, arising from the need to coordinate two separate components in one formulation poses significant challenges for establishing a reliable pharmacokinetic/pharmacodynamic (PK/PD) relationship, thereby complicating clinical translation [151].

In recent decades, attention has increasingly shifted toward nanovesicle-based delivery systems, which may overcome several limitations associated with both viral vectors and synthetic mRNA delivery platforms. These systems offer streamlined architectures capable of encapsulating and protecting therapeutic cargos while improving delivery efficiency [66,68,152]. Among them, EVs have emerged as particularly promising candidates. EVs are naturally derived nanoparticles with low immunogenicity and can be obtained from hepatocytes, liver stem cells, mesenchymal stem cells, and other cell sources [153,154]. Their intrinsic capacity to traverse biological barriers, engage with hepatic metabolic pathways, and carry diverse biomolecular cargos, including proteins, metabolites, small RNAs, and mRNA, positions them as compelling platforms for treating genetic liver disorders [155]. Supporting this concept, recent work has demonstrated that EVs can encapsulate RNA-based therapeutics to correct metabolic defects at the transcript level [156]. Earlier studies by Herrera et al. further illustrated that EVs secreted by human liver stem cells naturally carried argininosuccinate synthase 1 (ASS1), enabling transient correction of ASS1 deficiency in vitro [157].

However, the therapeutic utility of EVs is significantly influenced by their in vivo pharmacokinetics. Once administered systemically, EVs are rapidly cleared by the mononuclear phagocyte system in the liver and spleen, limiting their circulation time and reducing delivery efficiency. To address this, several strategies have been developed to enhance EV persistence in the bloodstream. Surface engineering to incorporate immune evasion signals is one approach; for example, expression of CD47, which interacts with SIRPα on macrophages, can extend EV circulation from approximately 30 min to nearly 2 h [158]. Similarly, coating EVs with membranes from cardiac resident macrophages increases immune evasion and prolongs circulation to 12–24 h. Modulation of clathrin heavy chain 1 (Cltc), which mediates endocytosis in the liver and spleen, also affects clearance. Cltc knockdown reduces MPS uptake and increases EV retention in target tissues, an effect demonstrated in doxorubicin-induced cardiomyopathy models where modified EVs showed reduced off-target sequestration [159].

Alternatively, blocking macrophage uptake pathways can prolong EV circulation without modifying the vesicles themselves. Pre-treatment with phosphatidylserine (PS) liposomes, Galectin-5 (Gal-5), or inhibitors of scavenger receptor class A (SR-A) reduces recognition and internalization of therapeutic EVs. For example, SR-A blockade using dextran sulfate decreases liver clearance and increases tumor directed EV accumulation up to threefold over 24 h [159].

Despite these advances, it is important to recognize that unmodified nanovesicles naturally accumulate in the liver, due to the unique anatomy and flow dynamics of hepatic sinusoids [66]. Blood entering the liver slows dramatically within sinusoidal networks that contain dense populations of resident macrophages, facilitating nanoparticle retention. Nanovesicles often adhere to sinusoid walls, and those smaller than ~200 nm can pass through fenestrated liver sinusoidal endothelial cells into the space of Disse, where hepatocytes and hepatic stellate cells may internalize them. Consequently, nanoparticles in the 6–200 nm range show preferential deposition in hepatic tissue. Understanding these cellular and anatomical interactions is essential for the rational design of nanovesicle-based therapies for liver disease, guiding decisions regarding vesicle size, surface composition, and targeting modifications.

Clinical trial evidence of the use of EVs on other systemic diseases have been extensively reviewed in the following publications [160,161] and support the translational potential of EV-based therapeutics for liver disease. In addition, preclinical studies of EVs as delivery vehicles in various disease conditions covered above highlight their applicability as potential delivery systems in a variety of acute and chronic liver diseases. Furthermore, their low immunogenicity and natural liver tropism make EVs viable future platform for hepatically targeted RNA or enzyme replacement therapies as well.

In addition to EVs, liposomes represent one of the most established nanovesicle platforms for therapeutic delivery and have been extensively used in both preclinical and clinical settings. Liposomes are spherical vesicles composed of phospholipid bilayers that can encapsulate hydrophilic molecules in their aqueous core and hydrophobic agents within the lipid membrane, offering a versatile structure for drug and nucleic acid delivery [22]. Their biocompatibility, customizable size, and ability to incorporate surface ligands for cell or tissue-specific targeting have made liposomes valuable tools in liver-directed therapies [111].

Liposomes can be engineered to enhance circulation stability and reduce immune recognition. PEGylation, the addition of polyethylene glycol chains to the liposomal surface, effectively creates a steric barrier that limits opsonization and slows clearance by the mononuclear phagocyte system. This modification extends plasma half-life and increases the likelihood of accumulation in target tissues [162]. However, prolonged PEG exposure can induce anti-PEG antibodies and accelerated blood clearance, motivating interest in newer stealth strategies such as zwitterionic coatings or biomimetic membranes [163].

Like EVs, liposomes also show preferential accumulation in the liver, driven by the organ’s highly permeable sinusoidal endothelium and the presence of Kupffer cells that regulate nanoparticle clearance [118]. Smaller liposomes (<100–150 nm) can pass through sinusoidal fenestrae and access hepatocytes within the space of Disse, enabling the delivery of mRNA, siRNA, and small molecule therapeutics directly to parenchymal liver cells [164]. For instance, lipid nanoparticle style ionizable liposomes have been successfully applied in mRNA-based therapeutics, including the clinical translation of mRNA vaccines and emerging treatments for metabolic liver diseases [165]. By tuning lipid composition, charge properties, and membrane rigidity, liposomes can be optimized to modulate biodistribution, intracellular uptake, and endosomal escape efficiency, key determinants for RNA-based correction of monogenic liver disorders.

Several clinically approved formulations, including liposomal doxorubicin and amphotericin B, provide a strong translational precedent, demonstrating the safety, manufacturability, and scalability of liposomal technologies [166]. Clinically validated liposomal formulations also highlight the feasibility of liposome-based hepatic therapy. PEGylated liposomal doxorubicin has demonstrated improved hepatic accumulation compared with free drug and remains widely used in oncology settings involving hepatic tumors [167]. Moreover, recent comparative analyses emphasize the stability, reproducibility, and translational readiness of liposomal architectures for liver-targeted drug delivery [152]. Overall, liposomes provide a modular and clinically validated platform for liver-targeted drug and gene delivery.

LNPs on the other hand, have been explored as nucleic acid delivery platforms. Early generation LNP formulations, however, were linked to notable liver toxicity in preclinical studies [168]. Moreover, recent findings have emphasized a key delivery barrier: only a limited fraction of the encapsulated mRNA successfully reaches the cytoplasm, highlighting the persistent challenge of inefficient endosomal escape [169]. Despite these hurdles, the field has progressed substantially, driven by sustained efforts to engineer LNPs with improved biocompatibility, stability, and delivery efficiency [28]. The widespread impact and clinical success of SARS-CoV-2 mRNA vaccines further accelerated global interest in LNP technology for therapeutic gene delivery [170]. Advances such as the development of next-generation ionizable lipids and the addition of targeting moieties like GalNAc have markedly improved LNP safety profiles and enhanced hepatocyte-specific uptake [55]. Preclinical studies have also indicated that repeated administration of LNPs is generally well tolerated, without eliciting harmful inflammatory or immune reactions [171]. Notably, co-delivery strategies combining LNP-packaged mRNA encoding the SB transposase with ssAAV donor templates have achieved robust genome modification in murine and non-human primate livers [172]. More recently, the emergence of precision genome-editing systems, including base and prime editors has further expanded interest in LNP-based in vivo editing approaches for liver-targeted therapeutic applications (https://clinicaltrials.gov/study/NCT06389877 accessed date: 10 November 2025).

Taken together, these advances position LNPs as a rapidly maturing platform capable of supporting both transient RNA delivery and durable genome correction in the liver. LNPs currently represent the leading clinically validated nanovesicle platform. The siRNA therapeutic Patisiran, delivered via LNPs, remains the most substantial proof of effective human liver-targeted RNA delivery, showing predominant hepatic uptake and favorable pharmacokinetics [173]. This milestone has catalyzed multiple clinical programs exploring LNP-based delivery of mRNA or siRNA for transthyretin amyloidosis, hypercholesterolemia, hemophilia, and metabolic liver diseases, positioning LNPs at the forefront of next-generation hepatic gene therapy.

However, translation of these approaches into broad clinical practice will require continued refinement in targeting specificity, long-term safety assessment, and large-scale manufacturing. The increasing convergence of lipid chemistry, RNA engineering, and precision genome editing suggests that LNPs will be at the heart of next-generation therapies aimed at metabolic and genetic disorders of the liver. As ongoing clinical trials begin to define safety parameters and therapeutic durability, LNP-based strategies are poised to transition from experimental proof-of-concept systems toward clinically actionable, disease-modifying.

From a public health point of view nanovesicles as delivery vehicles have various advantages and limitations. Nanovesicles have demonstrated efficacy in enhancing drug delivery and bioavailability while minimizing systemic toxicity and adverse effects, thereby offering substantial public health benefits. Liposomal formulations including Doxil^®^ [22], and Onivyde^®^ [174], which are currently employed in various cancer therapies, extend drug circulation time, facilitate targeted delivery, and decrease systemic toxicity compared to the unencapsulated form of these drugs. Additional examples, such as Abraxane^®^ [175], an albumin-bound paclitaxel nanoparticle, and the BNT162b2 vaccine (Pfizer-BioNTech), which employs lipid nanoparticles for mRNA delivery against COVID-19, further illustrate significant advancements in targeted drug administration. These innovations have contributed to improved quality of life, increased life expectancy, and reduced healthcare costs at both individual and public health system levels. The limitations of nanovesicles that influence public health as well have been included in Section 5 and Section 7 below. Ongoing evaluation and rigorous investigation of the potential health and safety impacts associated with engineered nanovesicle exposure are essential. Conducting dynamic, proactive, and ethically responsible research will advance the field as nanovesicles become increasingly significant and transformative within medicine and public health throughout the 21st century.

**Table 1:** Summary of preclinical studies of Nanovesicle Platforms for Liver Diseases.

Type	Incapsulated Drug	Preclinical Model	Therapeutic Outcome	References
**EV**	Native or engineered cargo (miR-122, miR-148a, miR-181a, miR-223, miR-27a, anti-TGFβ ASO, exoASO-STAT6)	Rodent models of hepatic fibrosis, NAFLD/NASH, HCC, HBV/HCV infection	Reduced inflammation and fibrosis. Reprogrammed macrophages. Enhanced hepatocyte regeneration. Antiviral and antitumor effects	[68,69,70,71,72,73,74,75,76,77,78,79]
**LNPs**	mRNA (hMUT, G6PC, OTC, PCSK9), siRNA, gene-editing components	Mouse and non-human primate models of metabolic liver disorders (MMA, GSDIa, OTCD), NAFLD, HCC	Restored enzyme activity. Normalized metabolic markers; Reduced hepatic steatosis. Improved survival and liver function. Efficient hepatocyte transfection	[92,93,94,95,96,97,98,99,100,101,102,103,104,105,106,107,108,109,110]
**Liposomes**	Small molecules and biologics: doxorubicin (Doxil^®^), curcumin, pirfenidone, silymarin; siRNA; interferon-α; antiviral drugs	Rodent models of HCC, viral hepatitis, NASH, liver fibrosis, acute liver injury	Increased hepatic accumulation and sustained release. Reduced systemic toxicity. Decreased inflammation and collagen deposition	[111,112,113,114,115,116,117,118,119,120,121,122,123,124,125,126,127,128,129,130,131,132]

## 5. Safety Considerations Across Nanovesicle Platforms

Pharmacokinetics, biodistribution, and safety profiles are fundamental to the rational design of nanovesicle-based therapies for liver disease. Following systemic administration, nanovesicles, lipid nanoparticles (LNPs), extracellular vesicles (EVs), and liposomes immediately interact with plasma proteins, forming a dynamic protein corona that profoundly reshapes their biological identity. This adsorbed protein layer governs nanoparticle recognition by hepatic cells and strongly influences their preferential routing toward hepatocytes versus Kupffer cells, thereby dictating both therapeutic uptake and off-target clearance. The central role of the protein corona in determining nanoparticle fate in vivo was first systematically demonstrated by Monopoli et al. [176], who showed that the biological behavior of nanoparticles is defined by their acquired biomolecular corona rather than by their synthetic surface alone. Subsequent studies confirmed that corona composition critically affects hepatic sequestration, macrophage recognition, and circulation half-life, ultimately controlling therapeutic efficacy and systemic toxicity [176].

LNPs, broadly used for siRNA and mRNA delivery, are efficiently internalized by hepatocytes through ApoE–LDLR–mediated mechanisms; however, their safety profile reflects a combination of acute immune activation and chronic hepatic effects. Acute reactions include the complement activation–related pseudo allergy (CARPA), characterized by flushing, chest tightness, and hypotension [177], and cytokine release and innate immune stimulation reported during mRNA–LNP administration [178]. Formulation-dependent chronic effects include low-grade hepatic inflammation, metabolic alterations, or complement priming. Next-generation ionizable lipids have been improved by optimizing pKa values, resulting in increased tolerability and hepatic transfection efficiency, as demonstrated by Hassett et al. [179].

Because of their endogenous membrane composition, EVs exhibit superior biocompatibility and minimal acute immunogenicity. Nevertheless, their PK profile is dominated by ultra-rapid clearance by liver and spleen macrophages [180], which severely limits their systemic exposure and therapeutic window. Chronic toxicity remains low, while biodistribution varies markedly depending on the cellular origin of EVs [181]. So far, engineering strategies like CD47 display, which prolong systemic circulation, have shown promise in early studies [182], although long-term safety data remain limited.

Among the earliest and clinically validated nanocarriers, liposomes display PK properties that are influenced by size, lipid composition, and surface chemistry. Early studies [183] demonstrated that PEGylation prolongs liposome circulation, though repeated dosing can trigger the ABC phenomenon mediated by anti-PEG IgM, described later by Ishida et al. [184]. Liposomes tend to naturally accumulate in the liver because of passive targeting; while this is advantageous in the context of hepatic diseases, it can reduce delivery efficiency to extrahepatic tissues and, upon chronic exposure, contribute to Kupffer cell saturation or mild lipid accumulation.

Taken together across all nanovesicle platforms, disease-modified liver architecture profoundly influences PK and safety. Poisson et al. [185] illustrated that fibrosis, steatosis, and chronic inflammation are associated with reduced sinusoidal fenestration, increased extracellular matrix deposition, and enhanced macrophage activation. These changes lead to poor access to hepatocytes, enhanced retention in fibrotic tissue, and amplified inflammatory responses [185].

Collectively, nanovesicle PK and safety profiles reflect a delicate interplay between protein corona formation, hepatic clearance mechanisms, immune activation, and underlying liver pathology. To translate these therapies into effective, safe, and predictable treatment for liver diseases, it is necessary to develop a specific mechanistic understanding of these platforms.

## 6. Nanovesicles and Personalized Medicine

According to the FDA, precision medicine is based on the concept that disease management should be tailored to the biological and environmental features of each individual, allowing treatments to be matched as closely as possible to patient-specific needs [186].

### 6.1. Surface Modification for Targeted Delivery

Surface functionalization through directional ligand conjugation represents one of the most advanced strategies to confer molecular targeting to lipid-based nanocarriers. As demonstrated by Jeevarathinam et al. [187], site-specific Fc-directed conjugation of monoclonal antibodies to lipid and polymeric nanoparticles enables high binding specificity while preserving physicochemical stability, thereby strongly supporting their application in targeted drug delivery and precision nanomedicine. More broadly, ligand attachment remains one of the most established approaches to achieve active targeting and cellular specificity in both liposomes and extracellular vesicles. In liposomal systems, the fluid nature of the lipid bilayer allows the dynamic organization of surface-anchored ligands, including antibodies, peptides, and aptamers, optimizing receptor binding and enhancing cell-specific uptake through receptor-mediated interactions [188]. Engineered EVs can be surface-modified through genetic fusion or chemical conjugation of targeting ligands to membrane proteins such as Lamp2b or tetraspanins, enabling precise receptor-driven targeting toward specific tissues, including brain, tumor, and liver cells [189]. These ligand-mediated strategies confer high cellular specificity and significantly improve therapeutic accumulation at target sites while minimizing off-target distribution. As demonstrated by Rampado et al., cloaking nanocarriers with biologically derived membranes enables the acquisition of self-recognition markers such as CD47, which inhibit phagocytic clearance and significantly prolong systemic circulation of the nanocarrier [190]. Importantly, membrane-coated nanoparticles can also inherit the natural tropism and homing properties of the source cells, enabling preferential accumulation in the liver and inflamed hepatic tissues while reducing off-target uptake [191]. This approach has been successfully applied in liver disease models, where red blood cell membrane-coated nanoparticles promoted liver regeneration and markedly reduced liver injury markers in acute hepatic failure, positioning membrane biomimicry as a highly promising strategy for precision and personalized liver-targeted drug delivery [191]. Beyond surface functionalization, the physicochemical properties of lipid-based nanocarriers and extracellular vesicles, namely particle size, surface charge (zeta potential), and lipid composition, play a pivotal role in dictating in vivo biodistribution, tissue accumulation, and systemic clearance [192]. Early PK studies demonstrated that smaller liposomes exhibit significantly prolonged circulation times compared with larger vesicles, which are more rapidly sequestered by the mononuclear phagocyte system, particularly in the liver and spleen. Similarly, surface charge strongly modulates biological fate: negatively charged vesicles are cleared more rapidly from the bloodstream, whereas near-neutral formulations display prolonged circulation and reduced macrophage uptake [192]. Lipid composition further acts as a critical determinant of clearance behavior, as enrichment in phosphatidylserine markedly accelerates hepatic uptake through macrophage-mediated recognition, whereas cholesterol incorporation stabilizes the membrane and extends circulation half-life. In parallel, recent advances in EV engineering demonstrate that controlled modulation of EV membrane composition and biophysical properties enables fine-tuning of intracellular trafficking, endosomal escape, and functional delivery efficiency in vivo, ultimately reshaping biodistribution and organ-specific accumulation [193].

### 6.2. Customizing the Therapeutic Cargo

Both liposomes and EVs exhibit a highly versatile loading capacity, enabling the encapsulation of a broad spectrum of therapeutic cargos, including small-molecule drugs, proteins, siRNA, mRNA, and DNA, thereby allowing flexible adaptation to different disease contexts [192]. In particular, EVs naturally transport diverse nucleic acids and bioactive molecules that directly reflect the physiological and pathological state of the parent cell, making their cargo intrinsically disease-adaptable [194]. The molecular composition of EV cargo, including tumor-specific DNA mutations, disease-associated miRNAs, and condition-dependent proteins, mirrors the molecular profile of the donor cells, highlighting the potential to tailor therapeutic payloads according to patient-specific genetic and molecular features. Moreover, both synthetic liposomes and engineered EVs can be deliberately loaded with customized therapeutic agents through physical, chemical, or biological methods, reinforcing their role as highly programmable platforms for precision and personalized nanomedicine [192]. Beyond drug and gene delivery, extracellular vesicles and lipid-based nanocarriers can be engineered as personalized immune modulators through the co-delivery of tumor-associated antigens and immune-stimulatory adjuvants [189]. Owing to their ability to present antigens in a native membrane context and to efficiently interact with antigen-presenting cells, EV-based platforms have shown strong potential for inducing patient-tailored anti-tumor immune responses while limiting systemic immune-related toxicity, thus supporting their application in personalized cancer immunotherapy [189]. At a more advanced level of customization, genetic engineering of parental cells represents a powerful strategy to generate extracellular vesicles that are intrinsically programmed with both therapeutic cargo and targeting functionalities. As extensively reviewed by Liu et al., donor cells can be transfected or transduced to overexpress specific nucleic acids, proteins, or membrane-associated ligands, which are subsequently incorporated into secreted EVs, yielding vesicles preloaded with therapeutic RNAs, proteins, or gene-editing systems [189]. Tang et al. further demonstrated that engineered EVs can be rationally designed to simultaneously carry customized therapeutic payloads and intrinsic homing properties derived from their parent cells, positioning genetically programmed EVs as flexible and highly tunable nanoplatforms for targeted and personalized nanomedicine [195].

### 6.3. Autologous Nanomedicine: Patient-Derived Platforms for Precision Therapy

As demonstrated by Shang et al. [196], EVs can be exploited both as disease biomarkers for early diagnosis and as a preventive strategy. Since EVs are present in virtually all body fluids and contain molecular signatures reflective of the originating cells, they represent a cornerstone of liquid biopsy approaches [196]. EV-based liquid biopsies are particularly promising for vulnerable populations in which invasive procedures pose significant risks, such as pregnancy, as well as for diseases that are notoriously difficult to detect at pre-symptomatic stages, including pancreatic cancer [197]. Beyond their diagnostic value, EVs display remarkable heterogeneity and plasticity, which strongly support their implementation in personalized medicine. In this context, the exploitation of patient-derived EVs may enable the development of highly individualized diagnostic and therapeutic interventions tailored to the specific molecular features of each patient [186]. For a truly personalized approach, EVs can also be directly derived from the patient’s own cells and bodily fluids, enabling autologous therapeutic strategies alongside patient-specific diagnostics in liver diseases. Autologous EVs exhibit intrinsic biocompatibility and markedly reduced immunogenicity while preserving native intercellular communication mechanisms, making them highly suitable as personalized therapeutic vectors [198]. In the hepatic context, systemically administered EVs naturally accumulate in the liver, and this tropism is further enhanced under conditions of liver injury, supporting their potential for tailored anti-fibrotic and regenerative therapies [198]. In parallel, the molecular analysis of EVs isolated from patient biofluids provides a robust platform for patient-specific diagnostics, as circulating EVs reflect the pathological state of their parent liver cells and carry disease-associated microRNAs, proteins, and nucleic acids that dynamically change with disease progression [199]. These features enable early, non-invasive detection, disease stratification, prognosis assessment, and real-time monitoring of therapeutic response in NAFLD, NASH, fibrosis, cirrhosis, and hepatocellular carcinoma. Notably, EV-enclosed microRNAs have emerged as particularly powerful patient-specific biomarkers for liver fibrosis; as comprehensively reviewed by Fagoonee et al., distinct panels of EV-associated miRNAs mirror both hepatocellular and cholestatic injury and closely correlate with fibrotic burden, reinforcing the role of EVs as personalized liquid biopsy tools for liver disease [200]. In parallel with naturally occurring vesicles, engineered extracellular vesicles can be exploited as customizable delivery platforms for precision therapeutic and diagnostic applications. Although their clinical translation is still largely confined to preclinical settings, engineered EVs can be efficiently loaded with therapeutic biomolecules or drugs and selectively directed toward specific tissues or cell types, thereby enhancing therapeutic efficacy while minimizing off-target distribution [201]. Alongside EV-based strategies, LNPs represent a highly adaptable platform for personalized nucleic acid delivery. As demonstrated by Jallow et al., LNPs can be optimized for highly specific, route-dependent, and tissue-selective RNA delivery, providing a powerful foundation for precision and patient-tailored RNA therapies across rare genetic disorders, cancer, and neurodegenerative diseases [202]. For example, the NANOSPRESSO platform [203] represents a paradigm shift toward tailored medicine by enabling on-demand, bedside production of patient-specific nucleic acid nanomedicines. By integrating microfluidic technology with lipid nanoparticle formulation, this system allows hospital pharmacists to locally manufacture RNA or gene-based therapies customized according to the individual patient’s genotype, disease mechanism, and therapeutic needs, overcoming the limitations of one-size-fits-all industrial manufacturing and offering a transformative solution for rare and orphan diseases. As regards liposomes, their high degree of tunability in lipid composition, size, surface charge, drug loading strategies, and surface functionalization enables the rational design of highly tailored nanocarriers for personalized medicine [204].

## 7. Limitations and Future Directions

Clinical translation of EVs requires addressing manufacturing, safety, and regulatory hurdles. Key challenges include establishing scalable Good Manufacturing Practice (GMP) processes, defining potency assays, and ensuring batch-to-batch consistency [205]. Regulatory agencies continue to emphasize the need for standardized isolation, characterization, and quality control methods. Several early-phase clinical trials, although not yet focused on genetic liver disorders, are evaluating EV-based therapeutics for cancer, immune modulation, and tissue repair [206]. These studies provide a foundational translational roadmap that can be adapted for liver-targeted applications. Importantly, the field benefits from prior experience in viral gene therapy, which underscores the importance of evaluating long-term safety, immune tolerance, and biodistribution patterns [207].

Despite this progress, significant translation barriers remain. Achieving consistent hepatocyte targeting, controlling cargo loading efficiency, and scaling EV production to clinically relevant volumes remain key challenges [208]. Additionally, robust preclinical models are needed to reliably predict therapeutic outcomes in humans [209]. Standardization of storage, dosing, and delivery route selection is critical to support regulatory approval and widespread clinical integration [208].

Looking ahead, a comparative view across nanovesicle platforms highlights complementary strengths and informs strategic development. Liposomes offer well-established clinical manufacturing pipelines and flexible chemical modification but can trigger immune clearance without careful surface engineering. LNPs, particularly next-generation ionizable formulations, have already demonstrated clinically validated delivery of RNA payloads and can be functionalized for hepatocyte-specific uptake; however, concerns regarding innate immune activation and repeated dosing must still be carefully managed. EVs, while intrinsically biocompatible and capable of engaging native hepatic signaling pathways, require advances in scalable production, reproducible cargo loading, and immune-evasion design.

Taken together, the future of liver-targeted nanovesicle therapeutics may rely not on choosing a single optimal platform but on integrating the advantages of each. Hybrid strategies, such as EV–LNP chimeras, EV surface engineering with liposomal lipids, or sequential EV/LNP delivery to optimize priming and editing, are emerging as promising avenues. Moreover, pairing these delivery systems with gene-editing technologies, including base and prime editors, holds potential for durable correction of monogenic liver diseases. Progress will ultimately depend on sustained collaboration between academic researchers, pharmaceutical developers, and regulatory bodies to ensure safe, reproducible, and scalable clinical translation [165].

## 8. Conclusions

In summary, nanovesicle-based systems represent a promising strategy for liver-targeted delivery of therapeutic cargos. EVs offer intrinsic biocompatibility and natural tropism, but challenges remain in scalable manufacturing, cargo loading, and pharmacokinetic control. Liposomes provides a clinically established platform with tunable surface chemistry, though their circulation time and immune interactions require careful optimization. LNPs have demonstrated robustness in vivo RNA delivery and continue to benefit from advances in ionizable lipid chemistry and hepatocyte-targeting ligands. No single system is universally optimal, and platform selection should be guided by therapeutic context, dosing requirements, and durability of effect. Future progress will likely arise from hybrid approaches that integrate the advantages of each vesicle type. Continued coordination between research, industry, and regulatory agencies will be essential to advance these platforms toward safe and effective clinical translation.

## Data Availability

No new data were created or analyzed in this study. Data sharing is not applicable to this article.

## Abbreviations

The following abbreviations are used in this manuscript:

LNPs	Lipid nanoparticles
EVs	Extracellular vesicles
mRNA	Messenger ribonucleic acid
siRNA	Small interfering ribonucleic acid
PEG	Polyethylene glycol
GalNAc	N-acetylgalactosamine
HSCs	Hepatic stellate cells
LSECs	Liver sinusoidal endothelial cells
